# Effect of partial substitution of chemical fertilizer with organic manure combined with biochar on lettuce yield, soil properties and leaf metabolism

**DOI:** 10.3389/fpls.2026.1858611

**Published:** 2026-06-17

**Authors:** Ping Li, Shixiong Li, Zonghao Li, Xilatu Dabu

**Affiliations:** Key Laboratory for Improving Quality and Productivity of Arable Land of Yunnan Province, College of Resources and Environment, Yunnan Agricultural University, Kunming, Yunnan, China

**Keywords:** biochar, lettuce, metabolomic profiling, organic substitution, quality, soil fertility

## Abstract

The co-application of biochar and organic fertilizer with chemical fertilizer is considered an important strategy for improving soil functions and vegetable quality. In this study, a field experiment was conducted with four treatments under equal total nutrient input: chemical fertilizer alone (T1), 50% substitution of chemical fertilizer nutrients with organic manure (T2), chemical fertilizer plus biochar (T3), and 50% substitution of chemical fertilizer nutrients with organic manure plus biochar (T4). The objective of this study was to investigate the effects of organic fertilizer and biochar application on lettuce yield, soil physicochemical properties, and leaf metabolomic profiles, thereby providing a scientific foundation for reducing chemical fertilizer inputs, enhancing fertilizer use efficiency, and improving vegetable quality. The results showed that, compared with T1, treatments T2, T3, and T4 increased soil organic matter, pH, available nitrogen, phosphorus, and potassium contents, as well as lettuce yield and nutrient uptake to varying degrees, with T4 exhibiting the most pronounced overall effects. Metabolomic analysis revealed that the application of organic fertilizer and biochar significantly up-regulated phenolic acids and flavonoids in lettuce leaves and promoted the enrichment of pathways associated with secondary metabolite accumulation, such as phenolic acid biosynthesis and sulfur metabolism. Correlation analysis further indicated that the changes in leaf metabolites were significantly correlated mainly with soil organic matter, pH, and available potassium. KEGG enrichment analysis uncovered differential impacts of the fertilization treatments on metabolic pathways: organic fertilizer primarily affected the tyrosine metabolism pathway, biochar mainly influenced nitrogen metabolism-related pathways (involving zeatin and amino acids), whereas the combination of organic fertilizer and biochar predominantly affected pathways including phenolic acid biosynthesis and sulfur metabolism. In conclusion, from a metabolomics perspective, this study revealed the metabolic pathway division of labor and synergistic effects of organic fertilizer and biochar in regulating secondary metabolism in lettuce, and preliminarily established associations between soil physicochemical factors and leaf metabolites, providing new insights into the quality improvement mechanism of organic substitution combined with biochar.

## 1 Introduction

Lettuce (*Lactuca sativa L.*), a variety of the species Lactuca sativa within the genus Lactuca of the Asteraceae family, is an annual herbaceous plant, also known as leaf lettuce (Xie et al., 2022). As a low-calorie, low-fat health food, leaf lettuce is rich in polyunsaturated fatty acids, dietary fiber, phenolic compounds, vitamin C, carotenoids, vitamin E, and other plant-derived health-promoting substances (Kim et al., 2016). It is also an important dietary source of trace elements, making it highly popular among consumers (Yang et al., 2018). Numerous studies have shown that it can improve cholesterol metabolism, reduce the risk of cardiovascular disease, and possess anticancer properties (Shi et al., 2022). However, intensive cultivation practices have led to issues such as biodiversity loss, reduction in soil organic matter, and deterioration of soil physical and chemical properties, resulting in decreased soil fertility and further impacting the nutritional quality and flavor characteristics of lettuce (Dincă et al., 2022). Therefore, exploring sustainable fertilization strategies to synergistically enhance both yield and secondary metabolite accumulation in lettuce has become an important direction in current lettuce cultivation research.

The partial substitution of chemical fertilizers with organic manure offers significant advantages in improving soil structure, enhancing nutrient use efficiency, and reducing environmental risks. Studies indicate that applying organic manure can increase soil organic matter, improve soil structure, enhance nutrient availability, and boost soil microbial activity, thereby significantly improving soil health and environmental quality and increasing crop productivity (Liu et al., 2024). However, the nutrient release rate of organic manure is relatively slow, and its sole application often cannot rapidly improve soil fertility and meet crop nutrient demand (Chatzistathis et al., 2021). Consequently, combining it with other amendment measures is necessary to optimize its effects. Biochar, a carbon-rich soil amendment, is widely used to enhance soil fertility, promote soil carbon sequestration, stimulate crop growth, and remediate contaminated soils, owing to its high alkalinity, porous structure, stable carbon content, and large surface area (Dai et al., 2021). Research has shown that the combined application of organic manure and biochar can promote soil physical and biochemical properties as well as plant productivity (Zhang et al., 2024).

In recent years, metabolomics techniques have provided an efficient means to elucidate the regulation of crop secondary metabolites by fertilization practices (Sun et al., 2024). Metabolomic studies have shown that organic manure mainly regulates flavonol metabolism, promoting the formation of compounds such as kaempferol and quercetin, thereby influencing antioxidant diversity and fruit coloration (Sun et al., 2025). Biochar-based fertilizers primarily modulate amino acid biosynthesis and flavonoid pathways, thereby increasing umami compounds like theanine and reducing bitter and astringent components such as catechins, ultimately improving tea flavor (Yang et al., 2025). These studies have laid an important foundation for understanding the relationship between fertilization and the synthesis of crop secondary metabolites. However, systematic comparisons of the effects of sole organic manure, sole biochar, and their combined application on the metabolic profiles of leafy vegetables, as well as elucidation of their functional differentiation and synergistic effects at the metabolic pathway level, remain relatively scarce. Furthermore, integrating soil physicochemical properties with crop secondary metabolites through correlation analysis to decipher the potential mechanisms by which fertilization regulates quality from the perspective of soil–crop interactions is also a weak link in current research. Lettuce, as a typical leafy vegetable, may exhibit composition and accumulation patterns of flavor-related secondary metabolites that differ from those of fruit crops, and the relevant regulatory pathways remain to be elucidated. In light of this, the present study conducted field experiments and employed widely targeted metabolomics to systematically analyze changes in the metabolic profiles of lettuce leaves, with the following objectives: (1) to evaluate the effects of different fertilization treatments on lettuce yield, nutrient uptake, and leaf secondary metabolite profiles; (2) to identify key differential metabolites and their enriched pathways, and to clarify the functional differentiation between organic manure and biochar at the metabolic pathway level; and (3) to analyze the associations between soil physicochemical properties and secondary metabolites in lettuce leaves. This study will provide new metabolic insights into the regulation of secondary metabolite accumulation in leafy vegetables through partial organic substitution combined with biochar, and will also serve as a scientific reference for optimizing nutrient management strategies in sustainable vegetable production.

## 2 Materials and methods

### 2.1 Experimental farm conditions

This experiment was carried out from August 23 to September 30, 2020, in Luxiying, Chengjiang City, Yuxi City, Yunnan Province (N24°39′6″, E102°54′48″), located within the Fuxian Lake Basin. The study area falls under a subtropical plateau monsoon climate zone, characterized by an average annual temperature ranging from 11.9 to 17.5 °C and annual precipitation of 900–1200 mm. The experimental soil was derived from a former vegetable field previously used for Chinese cabbage cultivation. Its initial physicochemical properties were as follows: pH 7.58, organic matter content 16.63 g/kg, available nitrogen 100.33 mg/kg, available phosphorus 10.9 mg/kg, and available potassium 164.33 mg/kg.

### 2.2 Materials

The tested variety was Italian lettuce(*Lactuca sativa* L.var. *ramosa* Hort), and the organic fertilizer was obtained by fermentation of tobacco waste as the main raw material (total nutrient ≥ 5%). The raw material of biochar was rice husk, and the pyrolysis temperature was 500 °C, pH 8.1, organic carbon 57.17%. Compound fertilizers (N-P_2_O_5_-K_2_O 17-17-17) were provided by Yunnan Weixin Agricultural Science and Technology Co., Ltd.

### 2.3 Experimental design

T1: chemical fertilizer alone, which is the local conventional fertilization technology; T2: 50% substitution of chemical fertilizer nutrients with organic manure; T3: chemical fertilizer plus biochar(The amount of biochar applied is equal to the volume of organic fertilizer in T2 treatment, and the application technology of chemical fertilizer is the same as that of T1.); T4: 50% substitution of chemical fertilizer nutrients with organic manure plus biochar. In this experiment, the nutrients of biochar were ignored. The experiment was conducted in a randomized block design with 4 treatments, 3 replicates per treatment and 12 plots.

Compound fertilizer was applied at a rate of 1067 kg/ha in three split applications via water dilution, with a base-to-topdressing ratio of 1:1:2. Organic fertilizer was applied as a basal dressing at a rate of 5441 kg/ha, incorporated once into the 0–20 cm soil layer. Biochar was applied as a basal dressing at a volume equivalent to that of the organic fertilizer, also incorporated once into the 0–20 cm soil layer.

The basal fertilizer was applied on August 23, 2020, followed by two topdressing applications on September 3 and September 14, 2020. Raised beds were manually prepared within each plot. Individual plots covered an area of 30 m², with each bed measuring 2.1 m in width and 13.5 m in length, a bed height of 10.0 cm, and a spacing of 20.0 cm between beds. Protective rows (20 m long × 1 m wide) were established around each plot. Perforated plastic film mulch was applied to each plot. One healthy seedling was transplanted per hole, with a planting density of 8 plants per row and 57 plants per column, resulting in a total of 456 plants per plot. All other cultivation and management practices followed local conventional methods.

### 2.4 Collection and determination of soil and plant samples

#### 2.4.1 Determination of soil physical and chemical properties

During the harvest period, a five-point sampling method was used to take 20 cm mixed soil samples from the tillage layer at each point. After natural air drying and grinding, the samples were placed in a 2-mm sieve in a marked sealed bag for subsequent determination of soil pH, organic matter and available nutrients. The soil pH (soil-water ratio of 1:2.5) was determined by potentiometric method, the soil organic matter content (SOM) was determined by potassium dichromate oxidation-external heating method, the soil available nitrogen content (AN) was determined by alkaline hydrolysis diffusion method, the soil available phosphorus content (AP) was determined by NaHCO_3_ extraction-molybdenum blue colorimetric method, and the soil available potassium content (AK) was determined by NH_4_OAc extraction-flame photometric method.

#### 2.4.2 Lettuce yield and total nutrient determination

Plant samples were collected at the harvest stage, with 10 lettuce plants randomly selected from each plot. To ensure accurate yield measurement, the fresh weight of the sampled plants was recorded immediately after collection. From each treatment, six plants were randomly chosen for nutrient content analysis. These plants were first subjected to enzyme deactivation at 105 °C for 15 minutes, then oven-dried at 65 °C until a constant weight was achieved. Subsequently, the dried plant materials were ground into a homogeneous powder. The total nitrogen (TN) content in the plant tissues was determined by H_2_SO_4_-H_2_O_2_ digestion distillation method. Total phosphorus (TP) was measured by the molybdenum yellow colorimetry method, and total potassium (TK) was analyzed by flame photometry.

### 2.5 Metabolomics detection of lettuce leaves

#### 2.5.1 Sample extraction procedure

Lettuce samples were lyophilized using a vacuum freeze-dryer (Scientz-100F), ground into a fine powder with a grinding mill (MM 400, Retsch) at 30 Hz for 1.5 min, and then 100 mg of the powder was weighed and dissolved in 1.2 mL of 70% aqueous methanol extraction solution. The mixture was vortexed for 30 seconds at 30-minute intervals, repeated six times in total. Samples were subsequently stored overnight at 4 °C, followed by centrifugation at 12,000 rpm for 10 minutes. The supernatant was collected, filtered through a 0.22 μm microporous membrane, and stored in injection vials for UPLC-MS/MS analysis.

#### 2.5.2 Ultra-performance liquid chromatography conditions

Chromatographic separation was performed on an Agilent SB-C18 column (1.8 µm, 2.1 mm × 100 mm). The mobile phase consisted of (A) ultrapure water with 0.1% formic acid and (B) acetonitrile with 0.1% formic acid. The gradient elution program was as follows: 0.00 min, 5% B; increased linearly to 95% B over 9.00 min, maintained at 95% B for 1.00 min; from 10.00 to 11.10 min, decreased to 5% B, and then equilibrated at 5% B until 14.00 min. The flow rate was set at 0.35 mL/min, the column temperature was maintained at 40 °C, and the injection volume was 5 µL.

#### 2.5.3 Mass spectrometry conditions

Linear ion trap (LIT) and triple quadrupole (QQQ) scans were performed using a triple quadrupole linear ion trap mass spectrometer (Q TRAP), specifically the AB4500 Q TRAP UPLC/MS/MS system, which was equipped with an ESI Turbo Ion-Spray interface and operated in both positive and negative ion modes under the control of Analyst 1.6.3 software (AB Sciex). The operating parameters of the ESI source, as well as the instrument tuning and mass calibration, were consistent with those described in a previously published study (Wu et al., 2024).

#### 2.5.4 Metabolomics data analysis and differential metabolite screening

Mass spectrometry data were processed using Analyst 1.6.3 software. Compound identification based on secondary mass spectra was performed using an in-house database, MWDB (Metware Database). Metabolite quantification was carried out in multiple reaction monitoring (MRM) mode with the QQQ-MS. Variable Importance in Projection (VIP) values were calculated using R software and the MetaboAnalystR package (v1.0.1). Orthogonal partial least squares-discriminant analysis (OPLS-DA) was conducted. Differential metabolites were screened based on the criteria of fold change (FC) ≥ 2 or ≤ 0.5 and VIP ≥ 1, and were functionally annotated using the KEGG database.

### 2.6 Statistical analysis methods

Data were processed using Excel 2019. Analysis of variance (ANOVA) was performed using SPSS 26.0. Figures were generated using Origin 2021 and R (Version 4.5.2). For metabolomics data analysis and correlation analysis, R packages including “randomForest”, “ggplot2”, “multcompView”, “tidyverse”, “dplyr”, “linkET”, and “vegan” were employed. Additionally, multiple comparisons between different treatments were performed using the Least Significant Difference (LSD) method (*P < 0.05*).

## 3 Results and analysis

### 3.1 Effects of partial substitution of chemical fertilizer with organic manure combined with biochar application on lettuce yield and total nutrient content

**Figure 1:**
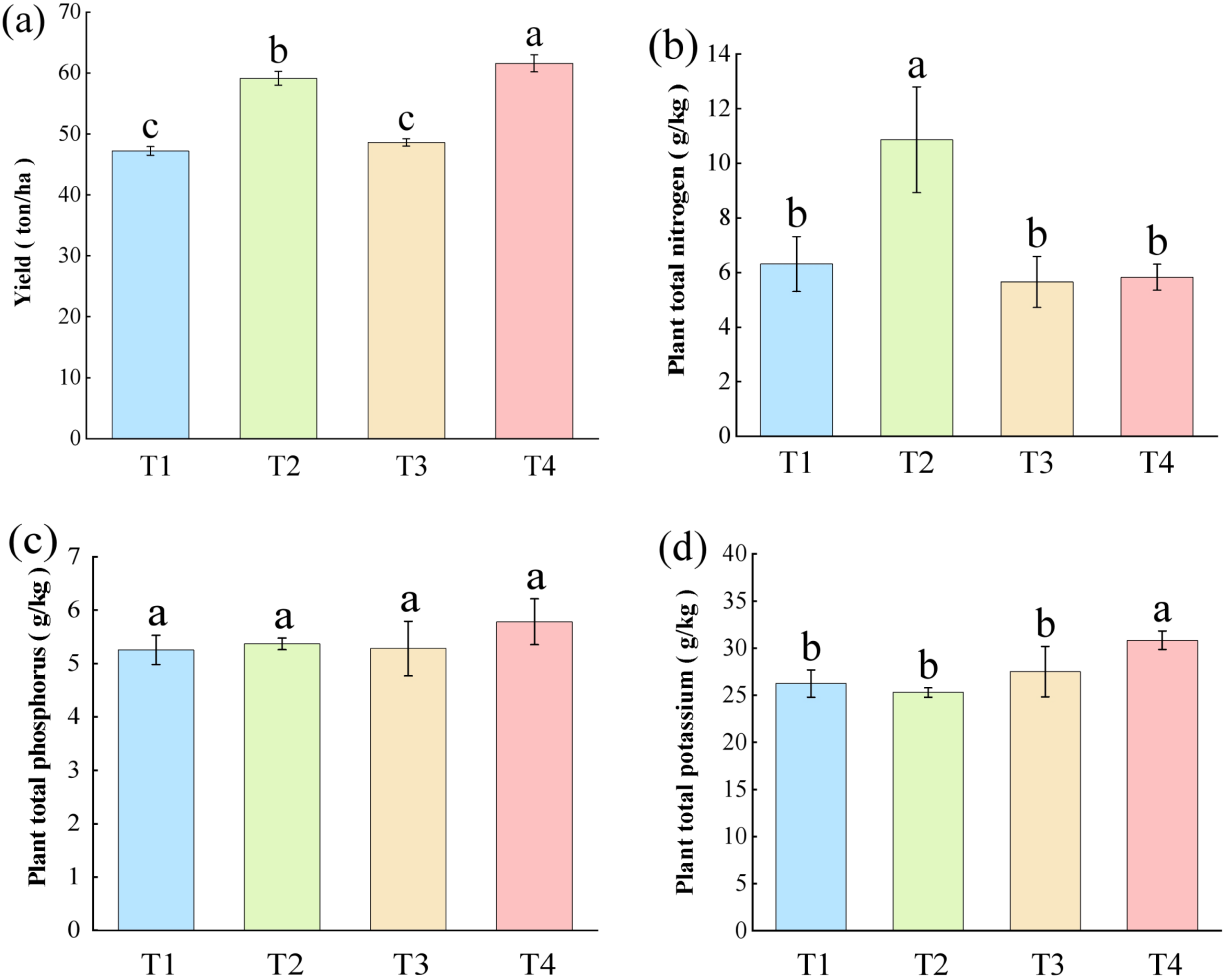
Effects of different treatments on lettuce yield and nutrient uptake. T1: chemical fertilizer alone; T2: 50% substitution of chemical fertilizer nutrients with organic manure; T3: chemical fertilizer plus biochar; T4: 50% substitution of chemical fertilizer nutrients with organic manure plus biochar. **(A)** Yield; **(B)** Plant total nitrogen content; **(C)** Plant total phosphorus content; **(D)** Plant total potassium content. Note: Different lowercase letters (a, b, c) in the figure indicate significant levels between different treatments (*P< 0.05*).

As shown in **Figure 1**, lettuce yield differed significantly among the fertilization treatments (*P < 0.05*). Compared with T1, yield increased significantly by 25.26% in T2 and by 30.48% in T4. Regarding total plant nutrients, the highest total phosphorus (TP) and total potassium (TK) contents were recorded in treatment T4. While no significant differences in TP content were observed among treatments, the TK content in T4 was significantly higher than in the other three treatments. Total nitrogen (TN) content followed the order T2 > T1 > T4 > T3, with T2 being significantly higher than all other treatments (*P < 0.05*). Compared to T1, T2 and T3, the TK content in T4 increased by 17.53%, 21.95% and 12.10%, respectively.

### 3.2 Effects of partial substitution of chemical fertilizer with organic manure combined with biochar on soil physical and chemical properties

**Figure 2:**
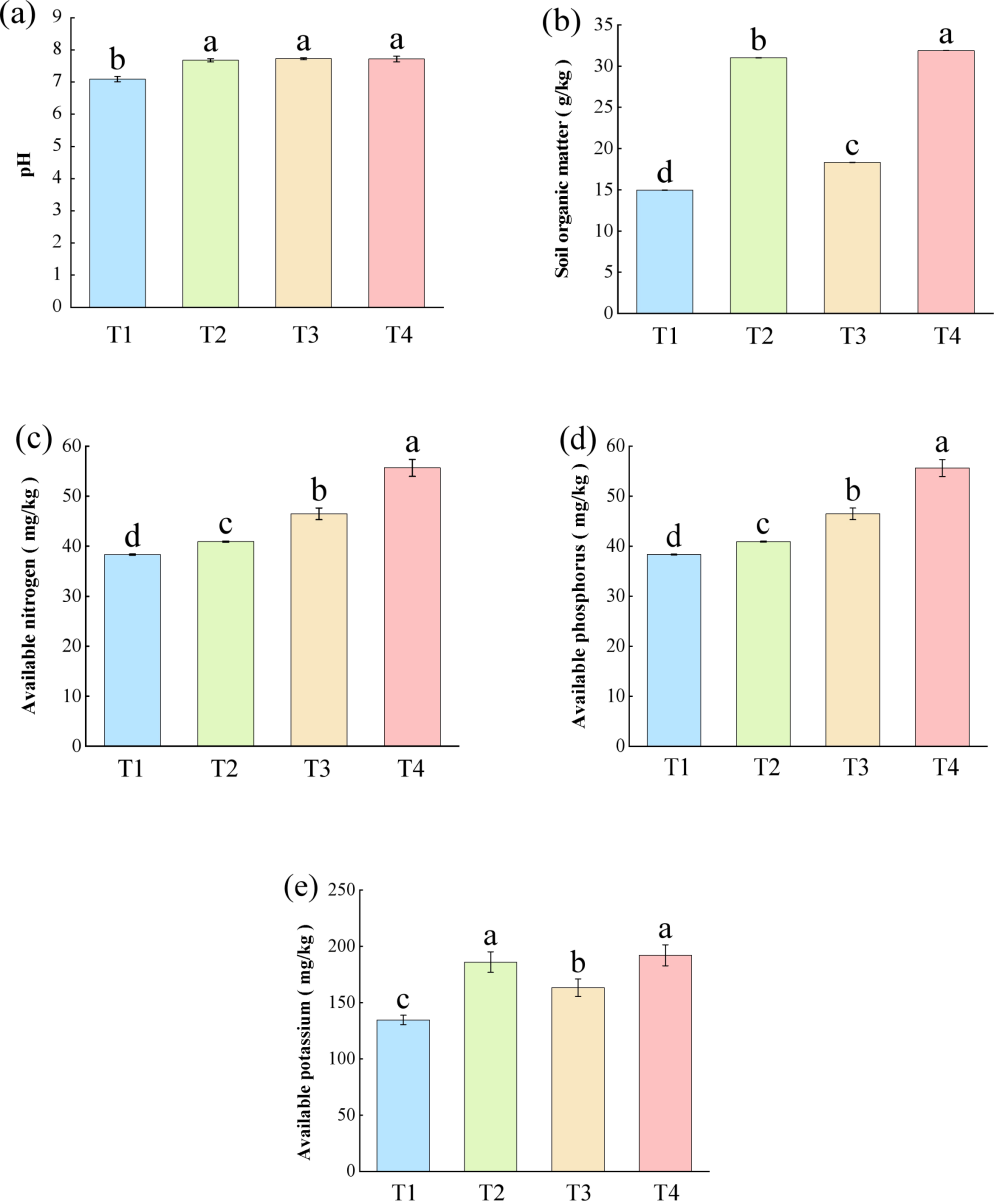
Effects of different treatments on soil physical and chemical properties. T1: chemical fertilizer alone; T2: 50% substitution of chemical fertilizer nutrients with organic manure; T3: chemical fertilizer plus biochar; T4: 50% substitution of chemical fertilizer nutrients with organic manure plus biochar. **(A)** pH; **(B)** Soil organic matter; **(C)** Available nitrogen; **(D)** Available phosphorus; **(E)** Available potassium. Different lowercase letters (a, b, c) in the figure indicate significant levels between different treatments (*P < 0.05*).

It can be seen from **Figure 2**, the soil *physical and chemical properties* under the T2, T3 and T4 treatments all differed significantly from those under the T1 treatment (*P < 0.05*). Among these, the highest pH value was recorded in treatment T3, followed by T4 and T2, with T1 being the lowest. The trend of change in soil organic matter content was similar to that of the available nutrient contents, with T4 showing the highest values, followed by T2 and T3, and T1 remaining the lowest. Among all fertilization treatments, T4 demonstrated the most effective improvement across all measured indicators. Compared to T1, the T2 treatment significantly increased soil organic matter (SOM), available nitrogen (AN), available phosphorus (AP), and available potassium (AK) by 51.81%, 9.49%, 6.73% and 38.20%, respectively (*P < 0.05*). Correspondingly, the T4 treatment resulted in significant increases of 53.11%, 19.70%, 45.19%, and 42.71% (*P < 0.05*) for these same parameters.

### 3.3 Effects of partial substitution of chemical fertilizer with organic fertilizer combined with biochar on leaf metabolism in lettuce

**Figure 3:**
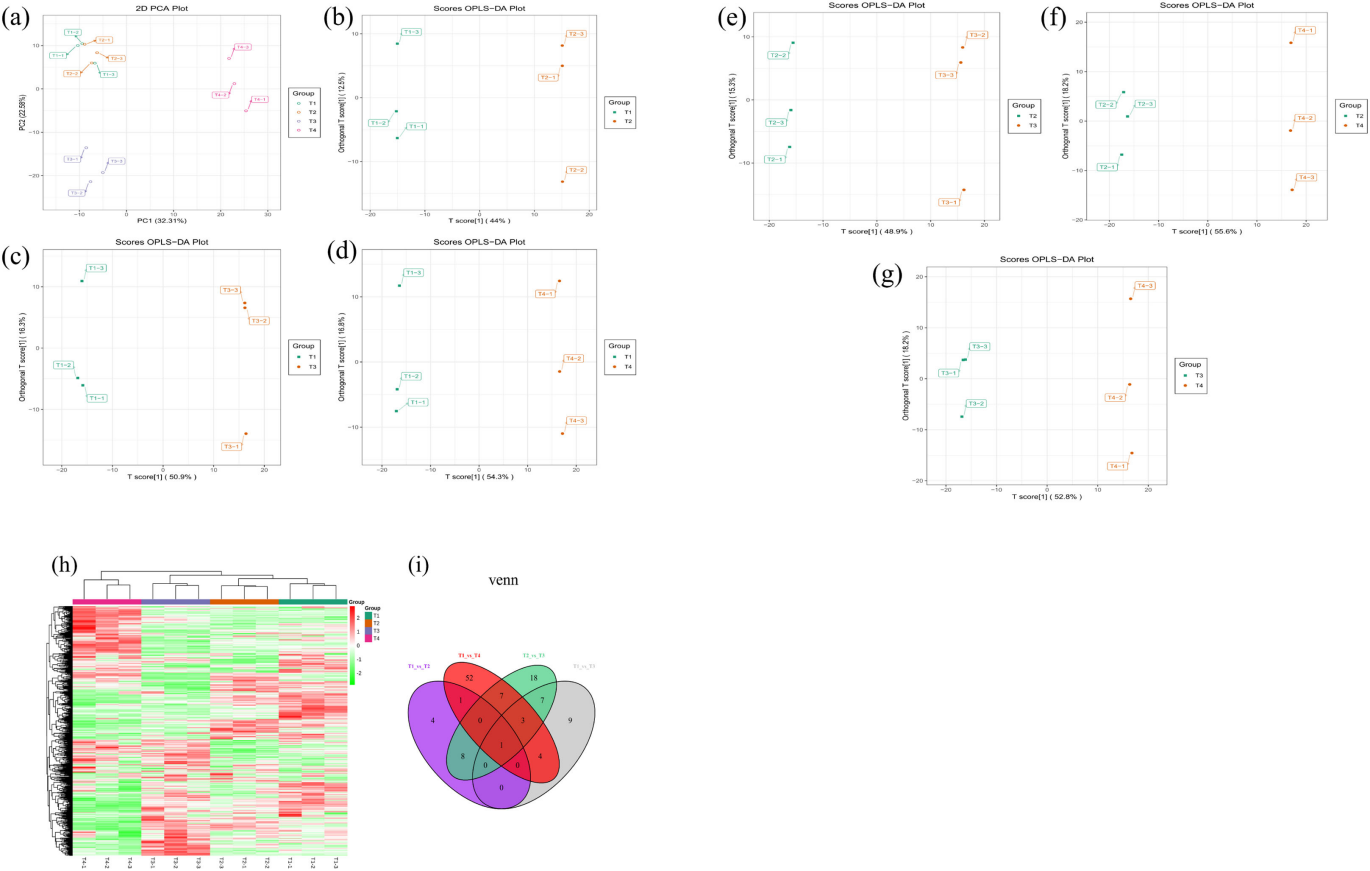
Effects of different fertilization treatments on leaf metabolism. T1: chemical fertilizer alone; T2: 50% substitution of chemical fertilizer nutrients with organic manure; T3: chemical fertilizer plus biochar; T4: 50% substitution of chemical fertilizer nutrients with organic manure plus biochar. **(A)** PCA analysis between different treatments: PC1 represents the first principal component, PC2 represents the second principal component, and the percentage represents the interpretation rate of the principal component to the data set; each point in the figure represents a sample, the samples of the same group are represented by the same color, and the Group is grouped (the same below); **(B–G)** Analysis of OPLS-DA between different treatments: the abscissa represents the predicted principal component, and the abscissa direction can see the gap between the groups; the ordinate represents the orthogonal principal component, and the ordinate direction can see the gap within the group; the percentage represents the component ‘s interpretation of the data set. Each point in the figure represents a sample, and the samples of the same group are represented by the same color. **(H)** Metabolite clustering heat map between different treatments: the horizontal is the sample name, the vertical is the metabolite information, the Class is the material classification, and the different colors are the values obtained after the relative content standardization treatment (red represents the high content, green represents the low content). The clustering line on the left side of the map is the metabolite clustering line, and the clustering line above the map is the sample clustering line; **(I)** Venn diagram of differential metabolites between different treatments: Each circle in the graph represents a comparison group. The numbers of circles and overlapping parts of circles represent the number of common differential metabolites between comparison groups, and the numbers without overlapping parts represent the number of unique differential metabolites between comparison groups.

Metabolomic analysis of lettuce leaf samples revealed the detection of a total of 622 metabolites, encompassing 11 categories including lipids (19.1%), phenolic acids (18.4%), flavonoids (13.5%), amino acids and their derivatives (11.4%), organic acids (10.1%) and alkaloids (5.7%). A Venn diagram illustrates the relationships among differential metabolites across the different fertilization treatments (**Figure 3I**). A total of 622 metabolites showed significant differences among the four comparison groups, with 11 common differential metabolites identified. The principal component analysis (PCA) results (**Figure 3A**) indicated that the metabolic data from different treatment groups demonstrated clear separation trends on the PCA score plot. The contribution rate of the first principal component (PC1) was 32.31%, and that of the second principal component (PC2) was 22.58%. Further orthogonal partial least squares-discriminant analysis (OPLS-DA) (**Figures 3B–G**) revealed distinct separation between the different comparison groups, confirming significant differences in lettuce metabolites under the various fertilization treatments. To intuitively display the changes in metabolite relative abundance, a cluster heatmap was generated for different metabolite categories across the sample groups (**Figure 3H**). The heatmap showed that metabolites exhibited distinct expression patterns among the different treatment groups, demonstrating that the treatments significantly altered the metabolite profile in lettuce leaves.

### 3.4 Effects of partial substitution of chemical fertilizer with organic manure combined with biochar on leaf metabolites in lettuce

To investigate the trends in relative content changes of metabolites across different groups, the average relative content of differential metabolites in each group was normalized using z-score transformation, followed by K-means clustering analysis. The clustering results indicated significant inter-group heterogeneity among the T1, T2, T3 and T4 sample groups. Hierarchical clustering analysis revealed that the metabolites in lettuce under the four different fertilization treatments were distinctly classified into three major clusters. Specifically, T1 and T2 clustered together, T2 and T3 formed another cluster, and T4 clustered independently. This suggests that the metabolite profiles in lettuce exhibited similar change trends between T1 and T2, as well as between T2 and T3, whereas the T4 treatment displayed a distinct trend compared to the other three fertilization treatments.

**Figure 4:**
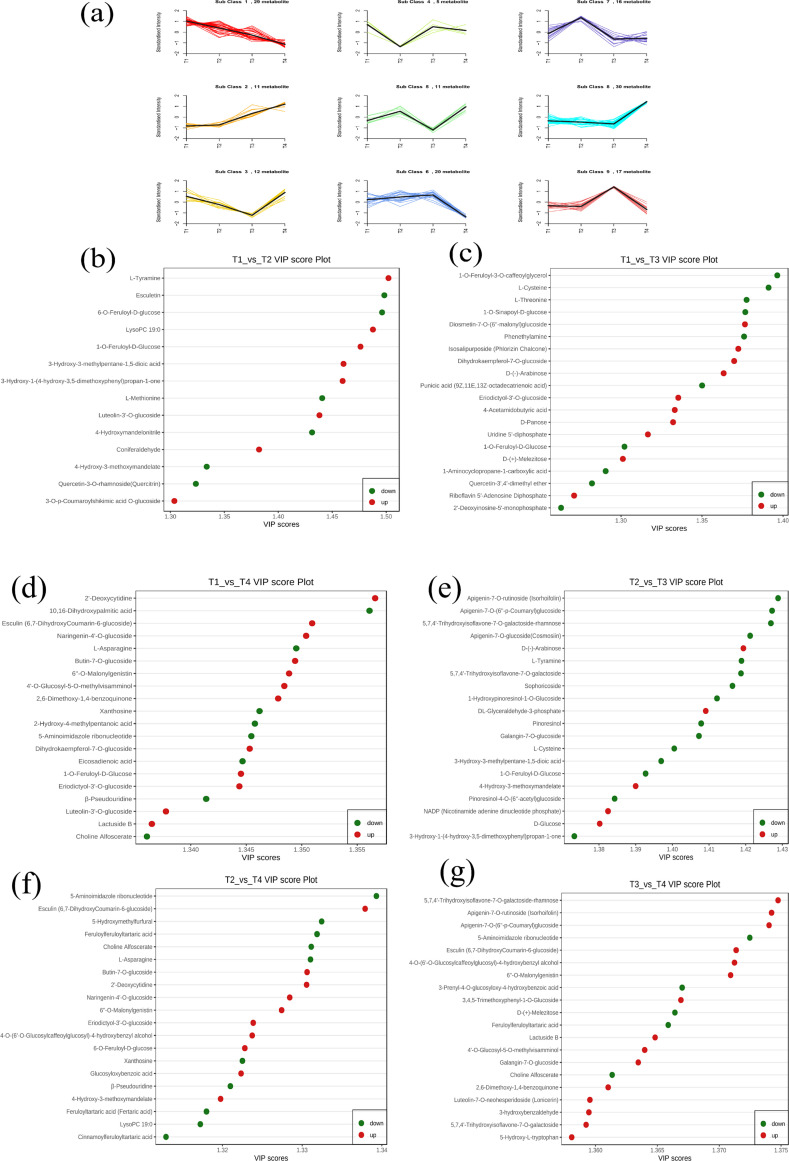
Effects of different treatments on metabolites in lettuce leaves. T1: chemical fertilizer alone; T2: 50% substitution of chemical fertilizer nutrients with organic manure; T3: chemical fertilizer plus biochar; T4: 50% substitution of chemical fertilizer nutrients with organic manure plus biochar. **(A)** K-means map of differential metabolites under different fertilization treatments: the abscissa represents the sample name, the ordinate represents the standardized relative content of metabolites, subclass represents the metabolite category number with the same change trend, and * metabolite represents the number of metabolites in this category is *; **(B–G)** VIP value diagram of differential metabolites under different fertilization treatments: abscissa represents VIP value, ordinate represents differential metabolites, red represents up-regulated differential metabolites, and green represents down-regulated differential metabolites.

Metabolites showing significant differences among the fertilization treatments were screened based on Variable Importance in Projection (VIP) values. As shown in **Figures 4B–G**, compared to T1, 14 differential metabolites were identified in the T2 treatment, of which 8 were up-regulated and 6 were down-regulated. The T3 treatment showed 24 differential metabolites compared to T1 (12 up-regulated, 12 down-regulated), and the T4 treatment showed 68 differential metabolites (30 up-regulated, 38 down-regulated). Compared to T2, the T3 treatment had 44 differential metabolites (15 up-regulated, 29 down-regulated), and the T4 treatment had 66 (31 up-regulated, 35 down-regulated). Compared to T3, the T4 treatment yielded 81 differential metabolites (44 up-regulated, 37 down-regulated).

In the T1_vs_T2 comparison group, phenolic acids exhibited the highest content and abundance, with most showing up-regulation and high VIP values. In the five comparison groups T1_vs_T3, T1_vs_T4, T2_vs_T3, T2_vs_T4, and T3_vs_T4, flavonoids displayed the highest content and abundance, with the majority being up-regulated and associated with high VIP values.

### 3.5 Effects of partial substitution of chemical fertilizer with organic fertilizer combined with biochar on leaf metabolic pathways in lettuce

**Figure 5:**
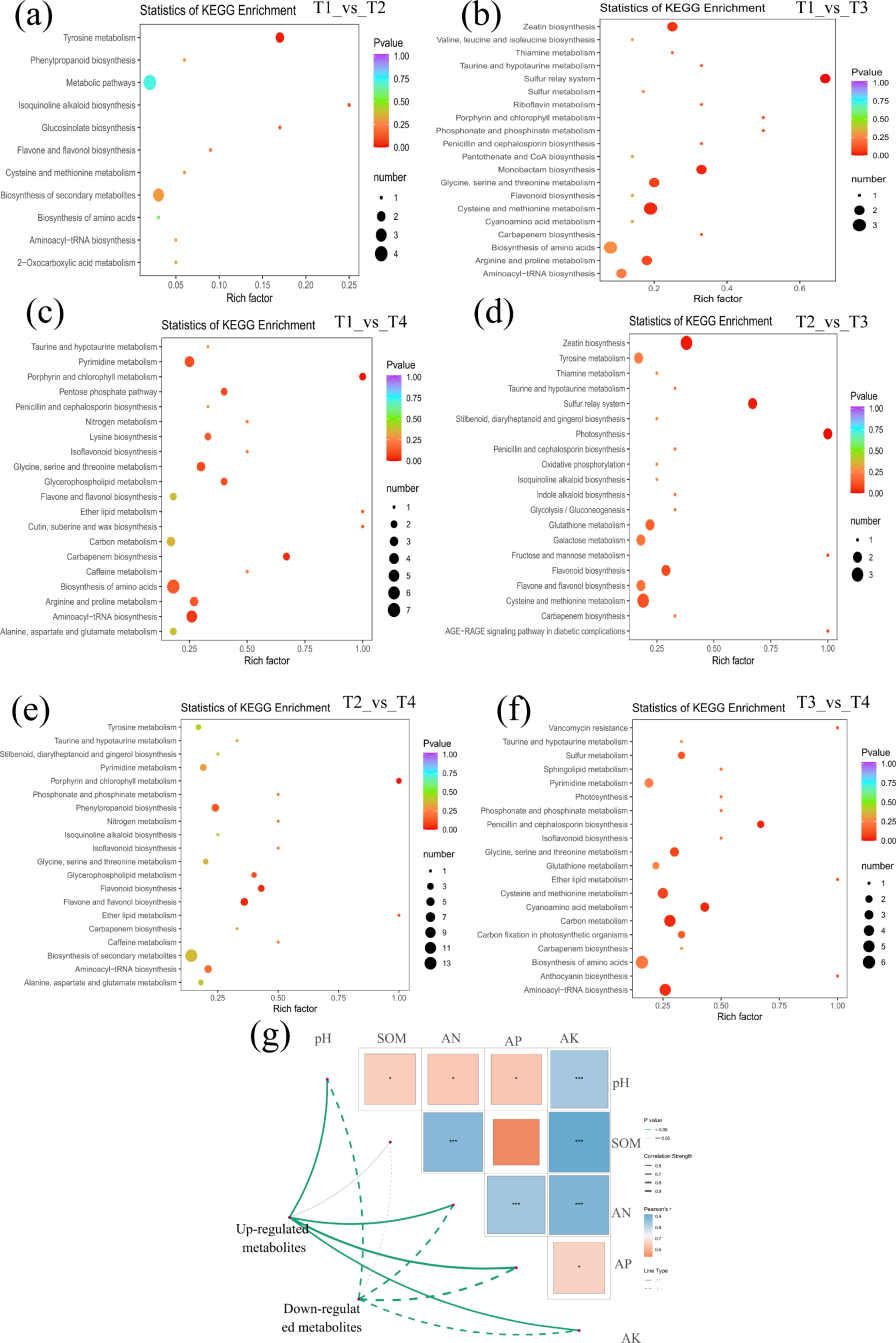
Effects of different treatments on metabolic pathways in lettuce leaves. T1: chemical fertilizer alone; T2: 50% substitution of chemical fertilizer nutrients with organic manure; T3: chemical fertilizer plus biochar; T4: 50% substitution of chemical fertilizer nutrients with organic manure plus biochar. The KEGG enrichment map of differential metabolites, the abscissa represents the Rich factor corresponding to each pathway, the ordinate is the pathway name, the color of the point is P-value, and the redder the more significant the enrichment. The size of the point represents the number of differential metabolites enriched **(A–F)**. Mantel test analysis **(G)**. In the figure **(G)**, SOM represents soil organic matter; AN represents available nitrogen; AP represents available phosphorus; AK represents available potassium.

Based on the differential metabolite screening results, the significantly different metabolites in lettuce were subjected to pathway analysis using the KEGG database. **Figures 5A–F** displays the top 20 enriched pathways for each comparison group. The KEGG enrichment bubble plots revealed the following patterns: in T1_vs_T2, the significantly enriched metabolic pathways primarily included tyrosine metabolism, biosynthesis of secondary metabolites, and isoquinoline alkaloid biosynthesis. In T1_vs_T3, the significantly enriched pathways were mainly biosynthesis of amino acids, zeatin biosynthesis, sulfur relay system, and cysteine and methionine metabolism. For T1_vs_T4, significant enrichment was observed in pathways such as biosynthesis of amino acids, pyrimidine metabolism, porphyrin and chlorophyll metabolism, pentose phosphate pathway, and lysine biosynthesis. In T2_vs_T3, the significantly enriched pathways included zeatin biosynthesis, sulfur relay system, flavonoid biosynthesis, and cysteine and methionine metabolism. The T2_vs_T4 comparison showed significant enrichment mainly in flavonoid biosynthesis, as well as flavone and flavonol biosynthesis pathways. In T3_vs_T4, the significantly enriched pathways were primarily sulfur metabolism, pyrimidine metabolism, and the biosynthesis of penicillins and cephalosporins.

Mantel test analysis (**Figure 5G**) indicated that among the differential metabolites, the up-regulated metabolites were primarily influenced by soil organic matter, while the down-regulated metabolites were affected by pH, organic matter and available potassium. These results suggest that up-regulated and down-regulated metabolites are driven by different environmental factors, implying potential complexity in the underlying regulatory mechanisms. Although the correlation analysis identified key soil factors, the precise biological mechanisms—whether these factors act directly or indirectly through altering microbial communities, root physiology or signaling pathways—require further in-depth investigation.

## 4 Discussion

### 4.1 Effects of partial substitution of chemical fertilizer with organic manure combined with biochar on soil physical and chemical properties

The results of this study indicated that, compared with chemical fertilizer alone, partial substitution of chemical fertilizer with organic manure, combined application of biochar with chemical fertilizer, and partial substitution of chemical fertilizer with organic manure combined with biochar all increased soil organic matter, pH, and available nutrient contents. Among these, the treatment involving partial substitution of chemical fertilizer with organic manure combined with biochar exhibited the most significant enhancement across all indicators. These findings are generally consistent with previous research. The coupling effect of biochar and organic manure exerts a greater positive impact on farmland ecology and soil physicochemical properties than the application of biochar or organic manure alone (Hu et al., 2024; Ngui et al., 2024). When biochar is applied to soil, the alkaline substances in biochar interact with soil minerals, thereby increasing soil pH. Additionally, functional groups on the biochar surface interact with soil active acids, buffering soil pH (Nkoh et al., 2021); consequently, the treatment combining chemical fertilizer with biochar showed the highest pH. In contrast, the treatment with 50% substitution of chemical fertilizer nutrients with organic manure may have produced small amounts of organic acids during organic manure decomposition, resulting in a weaker effect on pH increase compared with the chemical fertilizer plus biochar treatment. Some studies have also confirmed that organic manure application can effectively reduce the activity of metal ions such as K^+^, Na^+^, Ca²^+^, and Mg²^+^ in soil and lower soil pH. Moreover, organic manure provides nutrients for soil microorganisms, and the organic acids produced during microbial decomposition of organic matter can buffer or regulate the pH of alkaline soils (Shi et al., 2022). Regarding organic matter and available nutrients, the synergistic effect of the treatment with 50% substitution of chemical fertilizer nutrients with organic manure combined with biochar was most pronounced. On one hand, organic manure, as an exogenous organic material input, directly supplies organic carbon sources to the soil. Its application significantly increases the soil organic carbon stock, especially the stable organic carbon fraction associated with clay and silt particles, thereby enhancing total soil organic matter (Li et al., 2025b; Guo et al., 2019). On the other hand, the well-developed porous structure of biochar protects the habitat of soil microorganisms, thus facilitating their growth and reproduction, enhancing soil microbial activity, and promoting the mineralization and decomposition of soil organic carbon (Chen et al., 2018). Moreover, both materials are rich in organic carbon, promote soil aggregate formation, and provide physical protection for carbon, thereby achieving carbon sequestration (Yang et al., 2025). Our research group also found in experiments that organic manure increases active organic carbon, promotes aggregation and the formation of macroaggregates, thereby enhancing the active components of the carbon pool and nutrient supply capacity. Meanwhile, biochar, as an inert and stable carbon source, is directly sequestered in soil, enhancing the long-term stability of the carbon pool. Their combined application more effectively promotes the formation and stabilization of macroaggregates, improving soil nutrient status while enhancing the soil carbon pool (Peng et al., 2025). When biochar and organic manure are applied together, a synergistic effect occurs: organic manure provides sufficient readily degradable organic carbon and nutrients for microorganisms, while biochar stabilizes these substrates through its adsorption capacity and optimizes the microbial habitat. This interaction may activate soil enzyme systems, particularly phosphatases and proteases involved in key nutrient cycles. The protease-driven protein degradation process enhances the conversion of nitrogen to ammonium and nitrate nitrogen (nitrogen mineralization), while increased phosphatase activity promotes the transformation of organic phosphorus into plant-available inorganic phosphorus forms (phosphorus mobilization), thereby improving soil nutrient availability (Ali et al., 2025; Li et al., 2025a).

### 4.2 Effects of partial substitution of chemical fertilizer with organic manure combined with biochar on lettuce yield and total plant nutrient content

This study also found that partial substitution of chemical fertilizer with organic manure combined with biochar was the optimal treatment for achieving high lettuce yield and efficient potassium uptake, demonstrating the yield-increasing advantage of this combination. This may be because the input of organic manure provides stable and comprehensive nutrients for lettuce, improves soil aggregate structure and microbial activity, and creates a favorable environment for root growth. Meanwhile, biochar, with its large specific surface area and porous structure, further enhances soil water and nutrient retention capacity and promotes lettuce growth by improving the rhizosphere microenvironment. The synergistic effect of the two increases lettuce yield (Li et al., 2016). Regarding plant nutrient accumulation, this study found that the treatment with 50% substitution of chemical fertilizer nutrients with organic manure combined with biochar resulted in significantly higher total potassium content than all other treatments, indicating that biochar and organic manure played a positive role in promoting potassium uptake in lettuce. Research by Chen Yi et al. showed that the application of biochar-based fertilizers promoted nutrient uptake and accumulation in flue-cured tobacco and increased yield and agronomic efficiency (Chen et al., 2019). This may be due to the strong adsorption capacity of biochar for NH_4_^+^ and K^+^ in soil (Shujun et al., 2017; Zhao et al., 2021), combined with the fact that partial substitution with organic manure increases humic acid in the soil, which forms complexes with existing cations such as phosphorus and potassium, promoting the release of inorganic nutrients and increasing soil potassium content (Huang et al., 2024). Interestingly, this study found that the trend in total nitrogen content in lettuce plants was inconsistent with those of total phosphorus and total potassium, with the treatment involving 50% substitution of chemical fertilizer nutrients with organic manure showing the highest total nitrogen content, significantly higher than that of the treatment with 50% substitution combined with biochar. This suggests that partial substitution of chemical fertilizer with organic manure can more effectively promote nitrogen uptake and accumulation in lettuce. This may be because organic substitution regulates soil nitrogen transformation processes, providing exogenous carbon by enhancing microbial activity, increasing soil nitrogen fixation and organic carbon retention, thereby further improving crop yield and nitrogen use efficiency while reducing reactive nitrogen losses (Bi et al., 2022). This, in turn, enhances root activity and promotes nitrogen uptake in lettuce.

### 4.3 Effects of partial substitution of chemical fertilizer with organic manure combined with biochar on metabolites in lettuce

The flavor of lettuce primarily arises from the combined action of soluble solids such as sugars and organic acids, together with volatile flavor compounds including aldehydes, alcohols, ketones, and terpenes (Xu et al., 2023). These flavor substances are end products of both primary and secondary metabolic pathways in plants, and their accumulation levels are regulated by environmental factors and cultivation practices (Zhou et al., 2022). In this study, widely targeted metabolomics was used to systematically compare the effects of different fertilization treatments on the metabolic profiles of lettuce. It was found that different fertilization treatments had substantial effects on phenolic acids and flavonoids. Phenolic compounds are the most important endogenous antioxidants in lettuce, effectively scavenging free radicals and reducing oxidative damage. Studies have confirmed a significant positive correlation between the antioxidant capacity of lettuce and its total phenolic and total flavonoid contents, with key components such as chlorogenic acid, luteolin-7-O-glucuronide, and quercetin glycosides contributing most significantly to antioxidant activity (Yu et al., 2024). Recent studies have further validated significant positive correlations between total phenolic and total flavonoid contents and antioxidant indicators such as DPPH, ABTS, and FRAP across multiple lettuce varieties, providing evidence that phenolic compounds serve as the chemical basis for the antioxidant potential of lettuce (Moon et al., 2025). Compared with the sole application of chemical fertilizer, the majority of differential phenolic acids were up-regulated under 50% substitution of chemical fertilizer with organic manure, and many of these phenolic acids have been reported to possess antioxidant activity. From the perspective of metabolite accumulation, this suggests that partial organic substitution may favor the accumulation of compounds with potential antioxidant activity in lettuce, implying its potential to improve the nutritional quality and health value of lettuce. However, this inference still needs to be confirmed through direct antioxidant activity assays and sensory evaluation. Furthermore, compared with sole chemical fertilizer application, the treatments of chemical fertilizer plus biochar and 50% substitution of chemical fertilizer with organic manure plus biochar exhibited a higher proportion of flavonoids among the significantly differential metabolites, with most of these flavonoids being up-regulated. Moreover, the 50% organic manure substitution plus biochar treatment detected the highest number of differential metabolites, which may indicate that the co-application treatment has greater advantages in promoting the accumulation of flavonoids. These metabolites are closely associated with potential functions such as antioxidant activity and stress resistance (Isah, 2019). KEGG enrichment analysis revealed that the tyrosine metabolism pathway was significantly enriched in the comparison between chemical fertilizer alone and the 50% organic manure substitution treatment. Tyrosine is an important precursor for vitamin E (tocopherol) (Niu et al., 2022) and various alkaloids (Bhambhani et al., 2021), and serves as a starting point for many phenolic compounds (Xu et al., 2020). Vitamin E is a major lipophilic antioxidant in the cellular antioxidant system, protecting polyunsaturated fatty acids and cell membranes from free radical oxidation (Khallouki et al., 2022; Toro et al., 2024), while many alkaloids possess biological activities such as antibacterial and insecticidal properties, collectively enhancing plant stress resistance and adaptability. Additionally, studies have shown that tyrosine can be decarboxylated by tyrosine decarboxylase (TYDC) to produce tyramine, which is then hydroxylated by monophenol hydroxylase (MH) to produce dopamine (Liu et al., 2020). Dopamine, a water-soluble substance, has been demonstrated to possess potent antioxidant activity—higher than that of glutathione, catechin, flavonols, quercetin, and the flavone luteolin. Dopamine also influences plant sugar metabolism and cooperates with phytohormones to regulate plant growth (Liang et al., 2018). Transgenic studies have confirmed that overexpression of the tyrosine decarboxylase gene (TYDC) can significantly increase dopamine levels in plants, enhance antioxidant enzyme activities, and reduce the accumulation of reactive oxygen species(ROS) (Wang et al., 2020). This provides a molecular-level reference for understanding the putative connection between the enrichment of tyrosine metabolic pathways and the enhanced antioxidant potential in lettuce. Therefore, the addition of organic manure may activate the tyrosine metabolism pathway by improving the rhizosphere microenvironment and providing slow-release nutrients and signaling substances, thereby enhancing the health and antioxidant capacity of lettuce. The comparison results between chemical fertilizer alone and chemical fertilizer plus biochar, as well as between 50% organic manure substitution and chemical fertilizer plus biochar, both showed that the zeatin biosynthesis pathway was significantly enriched in the chemical fertilizer plus biochar treatment. This may be because biochar application affects plant physiology by regulating endogenous hormone levels. Research by Zhang Jie et al. indicated that exogenous zeatin application can increase the contents of osmotic regulatory substances such as soluble sugars, soluble proteins, and proline in pepper, as well as the activity of antioxidant enzymes, while reducing the accumulation of reactive oxygen species (Schäfer et al., 2015; Zhang et al., 2025), indirectly influencing the overall biosynthesis and accumulation of phenolic compounds.

The treatment with 50% substitution of chemical fertilizer with organic manure plus biochar formed an independent cluster in the cluster analysis. Compared with the 50% organic manure substitution treatment, the flavonoid biosynthesis pathway was significantly enriched in this treatment; meanwhile, compared with the chemical fertilizer plus biochar treatment, the sulfur metabolism pathway was also significantly enriched. These results suggest that the combined application of organic manure substitution and biochar may exert a synergistic promoting effect on flavonoid and sulfur metabolism. Sulfur supply level directly regulates the biosynthesis of glucosinolates, and adequate sulfur nutrition can significantly increase their content. These compounds also possess anticancer and antioxidant properties (Toro et al., 2024). Sulfur is an important component of sulfur-containing amino acids in plants, such as cysteine (Cys) and methionine (Met) (Wang et al., 2025). Furthermore, sulfur metabolism can facilitate the sulfation modification of phenolic compounds. Aryl sulfotransferases (ASTs) in sulfur metabolism catalyze the sulfation of flavonoids (e.g., quercetin, kaempferol) and phenolic acids, altering their solubility, stability, and biological activity, representing an important modification and detoxification pathway (Brodsky et al., 2024). Studies have shown that the application of organic manure and biochar can increase the abundance of soil microbial taxa related to the sulfur cycle (Hu et al., 2024). In biochar-amended soils, sulfur-mobilizing bacteria can convert organically bound sulfur into plant-available sulfate (SO_4_²^-^) by secreting arylsulfatase and other enzymes (Schmalenberger and Fox, 2016), and the enhanced microbial activity may promote the mineralization and availability of sulfur in the soil. Based on the above background, we speculate that organic substitution combined with biochar may promote the accumulation of sulfur-containing amino acids and sulfated phenolic/flavonoid compounds by stimulating sulfur-cycling microbial activity and enhancing rhizosphere sulfur availability, and this may represent one of the potential mechanisms underlying the pronounced changes in the secondary metabolite profile of lettuce under this treatment. However, this inference is currently based solely on metabolic pathway enrichment analysis; this study has not directly quantified soil sulfur forms, sulfur-cycling enzyme activities, or sulfated metabolites. Therefore, whether this hypothesis holds true remains to be further verified. As for whether the aforementioned changes in sulfur metabolism can further affect flavor-related substances, no definitive conclusion can be drawn, since volatile flavor compounds were not measured in the present experiment.

Flavonoid biosynthesis is a core pathway in plant secondary metabolism and a central branch of phenolic synthesis. Flavonoids represent the most important class of polyphenols. This pathway branches from the phenylpropanoid pathway to generate flavonols and other compounds, and its synthesis is precisely regulated by glycosyltransferases (UGTs) and MYB transcription factors (Cao et al., 2024). Studies have shown that the application of chemical fertilizer combined with biochar primarily affects the flavonoid biosynthesis pathway, promoting the conversion of naringenin to other flavonoids (e.g., apigenin, hesperetin), thereby enhancing the aroma, sweetness, and antioxidant capacity of tomatoes. In contrast, the application of organic manure alone or combined with biochar primarily affects the flavonol biosynthesis pathway, promoting the synthesis of flavonols such as kaempferol and quercetin, thereby enhancing the response of tomatoes to environmental stress (Chen et al., 2023; Dou et al., 2025; Sun et al., 2025). This is consistent with the significant up-regulation of flavonols observed under the organic substitution combined with biochar treatment in the present experiment.

Through Mantel test analysis, this study preliminarily established associations between soil physicochemical factors and changes in secondary metabolites in lettuce, providing clues for understanding the cascading relationship of “fertilization–soil–crop metabolism.” It should be noted that such correlation analysis only reveals correlations and cannot establish causality. Changes in soil factors may indirectly influence metabolism through pathways such as reshaping the rhizosphere microbial community and regulating root gene expression, which warrants further verification by integrating metagenomics and transcriptomics.

## 5 Conclusion

This study preliminarily investigated the effects of different fertilization treatments on soil physicochemical properties, lettuce yield, nutrient uptake, and leaf metabolites through a single-season field experiment. The results showed that both organic manure and biochar application improved soil physicochemical properties, lettuce yield, and secondary metabolites, with the combination of organic manure substitution plus biochar exhibiting the best overall performance. Metabolomic analysis revealed a clear pathway-level differentiation in the regulation of secondary metabolites in lettuce leaves by organic manure and biochar. Sole organic manure primarily upregulated phenolic acids and enriched the tyrosine metabolism pathway; sole biochar mainly enriched nitrogen metabolism-related pathways (zeatin and amino acid biosynthesis); whereas the combined treatment specifically enriched the phenolic acid biosynthesis and sulfur metabolism pathways, and significantly upregulated flavonoids, such as quercetin, naringenin, luteolin, kaempferol, and eriodictyol. This division of labor and synergistic effect at the metabolic pathway level constitutes the core metabolic finding of this study. Mantel test analysis indicated that changes in lettuce leaf metabolites were mainly significantly correlated with soil organic matter, pH, and available potassium, providing preliminary clues for understanding the cascading relationship of “fertilization–soil–crop metabolism”. In summary, the combined application of organic manure substitution and biochar can effectively improve soil nutrient supply, upregulate phenolic acid and flavonoid metabolites with antioxidant potential in lettuce leaves, and exhibit a division of labor and synergistic effect at the metabolic pathway level, thereby providing a metabolic-level scientific reference for optimizing fertilization strategies for high-quality leafy vegetable cultivation.

## 6 Research limitations and prospects

This study has the following limitations: (1) The field experiment was conducted only in a single season and at a single site. Due to the influence of climate, soil type, and interannual variability, the spatiotemporal generalizability of the findings should be cautiously extrapolated. Future studies should conduct multi-year and multi-site replicated trials in different ecological regions to verify the environmental robustness of the core metabolic pathways identified in this study, such as sulfur metabolism, tyrosine metabolism, and flavonoid biosynthesis pathways. (2) Each treatment in this experiment had only three biological replicates. Although this design is considered an acceptable minimum standard in metabolomics studies and the OPLS-DA model demonstrated significant between-group separation, increasing the number of replicates would further enhance statistical power, particularly for the detection of low-abundance differential metabolites. (3) The Mantel test analysis revealed significant correlations between soil organic matter, pH, available potassium, and secondary metabolites in lettuce leaves; however, such correlations cannot establish causality. Improvements in soil physicochemical properties may indirectly regulate plant metabolism by altering the structure and function of rhizosphere microbial communities. For instance, it remains unclear whether the enrichment of the sulfur metabolism pathway observed in this study is directly related to changes in the abundance of specific sulfur-oxidizing bacteria. Similarly, whether the upregulation of the flavonoid biosynthesis pathway involves changes in the expression of MYB transcription factors in plant roots also requires transcriptomic evidence.

Therefore, future research should integrate metagenomics and transcriptomics to construct a complete regulatory network of “soil microorganisms — root gene expression — leaf metabolites”, in order to systematically elucidate the microbial and molecular mechanisms by which the combined application of organic manure and biochar enhances the accumulation of secondary metabolites in leafy vegetables.

## Data Availability

The raw data supporting the conclusions of this article will be made available by the authors, without undue reservation.
